# Transactivation Assays to Assess Canine and Rodent Pregnane X Receptor (PXR) and Constitutive Androstane Receptor (CAR) Activation

**DOI:** 10.1371/journal.pone.0164642

**Published:** 2016-10-12

**Authors:** Marija Pinne, Elsa Ponce, Judy L. Raucy

**Affiliations:** Puracyp, Inc., Carlsbad, California, United States of America; Southern Illinois University School of Medicine, UNITED STATES

## Abstract

The pregnane X receptor (PXR/SXR, NR1I2) and constitutive androstane receptor (CAR, NR1I3) are nuclear receptors (NRs) involved in the regulation of many genes including cytochrome P450 enzymes (CYPs) and transporters important in metabolism and uptake of both endogenous substrates and xenobiotics. Activation of these receptors can lead to adverse drug effects as well as drug-drug interactions. Depending on which nuclear receptor is activated will determine which adverse effect could occur, making identification important. Screening for NR activation by New Molecular Entities (NMEs) using cell-based transactivation assays is the singular high throughput method currently available for identifying the activation of a particular NR. Moreover, screening for species-specific NR activation can minimize the use of animals in drug development and toxicology studies. With this in mind, we have developed *in vitro* transactivation assays to identify compounds that activate canine and rat PXR and CAR3. We found differences in specificity for canine and rat PXR, with the best activator for canine PXR being 10 μM SR12813 (60.1 ± 3.1-fold) and for rat PXR, 10 μM dexamethasone (60.9 ± 8.4 fold). Of the 19 test agents examined, 10 and 9 significantly activated rat and canine PXR at varying degrees, respectively. In contrast, 5 compounds exhibited statistically significant activation of rat CAR3 and 4 activated the canine receptor. For canine CAR3, 50 μM artemisinin proved to be the best activator (7.3 ± 1.8 and 10.5 ± 2.2 fold) while clotrimazole (10 μM) was the primary activator of the rat variant (13.7 ± 0.8 and 26.9 ± 1.3 fold). Results from these studies demonstrated that cell-based transactivation assays can detect species-specific activators and revealed that PXR was activated by at least twice as many compounds as was CAR3, suggesting that there are many more agonists for PXR than CAR.

## Introduction

Various nuclear receptors have been implicated in gene regulation of hepatic cytochrome P450 enzymes and transporters [1] including the glucocorticoid receptor, vitamin D receptor, constitutive androstane receptor (CAR) and pregnane X receptor (PXR); all of which are regulated by endobiotics (such as steroid hormones) and many different xenobiotics. Among these receptors, both CAR and PXR control the expression of CYP3A by xenobiotic exposure in animals and humans [2] through cross-talk mechanisms [1], making it difficult to identify which nuclear receptor is activated. The importance in identifying the NR being activated lies more in the adverse effects generated by such activation than with drug-drug interactions (DDIs). For example, PXR activation in transgenic mice can cause disruption of glucocorticoid and mineralocorticoid homeostasis [3] while activation of human CAR in these mice does not produce such an effect [3]. PXR activation also increases lipid synthesis and uptake in the liver producing disorders such as hepatic steatosis [4]. The mechanism involved appears to be an increased expression of human stearoyl-coenzyme A desaturase 1 and other lipogenic enzymes and transporters [4] leading to enhanced triglyceride accumulation and higher levels of total cholesterol [5]. In contrast, CAR activation exerts a beneficial effect by suppressing lipogenic gene expression and reducing serum triglyceride levels and very low density lipoprotein (VLDL) secretion from the liver. Another disorder in which PXR and CAR may exert opposing effects is type II diabetes [6]. The CAR agonist, phenobarbital, was shown to decrease plasma glucose levels and improve insulin sensitivity in diabetic patients [6]. However, individuals treated with the PXR agonist, rifampicin developed hyperglycemia [7]. This suggests that a PXR activator may produce an adverse effect while a CAR activator may be beneficial, emphasizing the importance of identifying the NR activated.

Whether PXR and/or CAR are activated by a compound stems from the ligand binding domain (LBD). This region is the most heterogenous region in NR sequences across vertebrate species and hPXR exhibits greater sequence diversity when compared to hCAR [8]. For CAR, significant heterogeneity is observed within the ligand-binding pocket (LBP) [8–10], which is normally more conserved than the rest of the LBD. With regards to PXR, there is a pronounced diversity within the LBP as witnessed by chemically unrelated compounds binding in the micromolar range [11]. Whether this holds true for animal PXR and/or CAR is not clear, but recent studies suggest a higher degree of compound specificity with rodent PXR when compared to human PXR [12].

While drug-drug interactions are not a major concern in the veterinary field, several animal species, particularly rodents are widely used to predict DDIs and adverse effects in humans and therefore it is crucial to elucidate species-specific differences in their activation. Furthermore, activation of animal NRs has implications in toxicology studies [13]. Screening for species-specific receptor activation can minimize the use of animals and aid in identification of complications associated with drug metabolism and clearance that may occur during pharmacokinetic analyses. An example of such complications would be cyp3a auto-induction reducing the exposure to the test article and rendering the safety evaluation ineffective. Taken together, this accentuates the need for simple and efficient screening methods for species-specific NR activation.

Here, we describe novel *in vitro* assays for high throughput screening (HTS) of canine and rat PXR and CAR. Importantly, we describe a cell based transactivation assay of canine PXR for the first time. Canine and rat CAR transactivation assays can be performed with either CYP2B6 or CYP3A4 response elements broadening the spectrum of utility. We demonstrate the species-specificity and report several chemicals that have not previously been shown to activate canine and rat PXR or CAR. Overall, the focus of this study was to address the need for assays that identify reagents that activate PXR and/or CAR in two separate species that play important roles in toxicological evaluations for safety and in overall drug development. Determining which receptor is being activated by an agent, can direct human investigations by supplying a mechanistic understanding of adverse drug effects produced via activation of either CAR or PXR.

Only *in vitro* systems such as those described here that employ a single receptor, can identify the exact mechanism for induction of specific genes.

## Materials and Methods

### Reagents and cell lines

DMSO was obtained from Amresco (Solon, OH). Clotrimazole, dexamethasone, artemisinin, miconazole, bis (2-ethylhexyl) phthalate (DEHP), ritanovir, efavirenz, omeprazole (OMP), α-naphthoflavone (ANF), phenobarbital sodium salt, phenytoin, 6-(4-Chlorophenyl)imidazo[2,1-b][1,3]thiazole-5-carbaldehyde O-(3,4-dichlorobenzyl)oxime (CITCO), 1,4-Bis-[2-(3,5-dichloropyridyloxy)]benzene, 3,3′,5,5′-Tetrachloro-1,4-bis(pyridyloxy)benzene (TCPOBOP), felodipine, forskolin, pregnenolone-16 α -carbonitrile (PCN) were purchased from Sigma-Aldrich (St. Louis, MO). Rifampicin, tetraethyl 2-(3,5-di-*tert*-butyl-4-hydroxyphenyl)ethenyl-1,1-bisphosphonate (SR12813) and troleandomycin were obtained from Enzo Life Sciences (Farmingdale, NY). Phusion high-fidelity DNA polymerase and “Hyclone” fetal bovine serum were purchased from Thermo Fisher Scientific (Waltham, MA). Cloning reagents and DMEM culture medium were purchased from Life Technologies (Carslbad, CA). QuikChange Lightning mutagenesis kit was obtained from Agilent Technologies (Santa Clara, CA). Canine liver cDNA was purchased from Zyagen (San Diego, CA). ONE-Glo Luciferase assay, CellTiter-Fluor cell viability assay components and Go-Taq polymerase were purchased from Promega (Madison, WI).

Human hepatoma cell line, HepG2 was obtained from American Type Culture Collection (Rockville, MD) and maintained in DMEM supplemented with 10% FBS, 100 U/ml penicillin and 100 μg /ml streptomycin at 37°C, 5% CO_2_. The stable cell line, rPXR, harboring rat PXR and the CYP3A4 promoter and enhancer linked to luciferase was produced and maintained as described previously [14].

### Plasmid constructs

To generate canine PXR (Accession number: XM_535750.3) with a start codon replicating that of human PXR (Accession number: AY091855), a *Canis lupus familiaris* liver cDNA was employed as the template. PCR conditions were 98°C for 2 min, 35 cycles of 98°C for 1 min, 59°C for 1 min and 72°C for 1 min 45 sec, followed by 72°C for 7 min and cooling to 4°C. The resulting PCR product was cloned into the pCR2.1 vector (Life Technologies, Carlsbad, CA) for sequence analysis and found to match that described in the NCBI Pubmed database. The correct product was cloned into an expression vector as previously described (Yueh et al., 2005).

To construct an expression vector harboring canine CAR3, reference CAR sequence (CAR1) for canine (Accession number: FJ202015) was amplified from *C*. *familiaris* liver cDNA. PCR conditions were 98°C for 2 min, 30 cycles of 98°C for 30 sec and 72°C for 1 min, followed by 72°C for 7 min and cooling to 4°C. The resulting PCR product was cloned into the pCR2.1 vector for sequence analysis and subsequently cloned into a pCDNA 3.1 expression vector (Life Technologies, Carlsbad, CA). The plasmid containing correct canine CAR1 was found to match that in the NCBI Pubmed database. Canine CAR1 was mutated to obtain CAR3 by introducing CTCCCTATCTTACA motif [15]. This was performed using forward primer: 5’- cctcttctctccggctccctatcttacagacaggcctggggtt -3’ and reverse primer: 5’- aaccccaggcctgtctgtaagatagggagccggagagaagagg -3’ in concert with QuikChange Lightning mutagenesis kit (Agilent) according to manufacturer’s instructions. The presence of correct canine CAR3 was verified by sequence analysis. CYP2B6 luciferase reporter vector containing the XREM-PBREM promoter and enhancer was obtained from Dr. Omiecinski (Penn State University) and has been described previously [16, 17]. To generate rat CAR3, a plasmid containing reference CAR (CAR1) [17] underwent mutagenesis as described above using 5’- gctctcttctctcctgctccctatcttacagacaggcctggggtt -3’ as the forward primer and 5’- aaccccaggcctgtctgtaagatagggagcaggagagaagagagc -3’ as the reverse primer. The incorporation of the human CAR3 motif into rat CAR matched that previously reported (Omiecinski et al., 2011). Firefly luciferase reporter construct harboring CYP3A4 proximal and distal enhancer regions has been described earlier [18]. Human RXRα coding region preceded by Kozak consensus sequence [19] was generated and the sequence found to match that previously described [16].

### Transient transfection of human hepatoma cells

HepG2 cells were seeded in 10-cm cell culture dishes and incubated until 60–70% confluency (about 5 days). For transfections, dishes were rinsed with phosphate buffered saline (PBS) and cells incubated for 1–4 h in OptiMEM (Life Technologies). LipofectAMINE 2000 (Life Technologies) was used at a 2:1 ratio (μl of transfection reagent to μg of DNA). For transfections involving canine and rat CAR3, 24 μg of total DNA was used (9 μg of luciferase reporter construct, 7.5 μg of either empty expression vector or CAR expression construct and 7.5 μg of the hRXRα construct). For transfections with the canine PXR, 21 μg of total DNA was employed (12 μg of luciferase reporter containing CYP3A4 proximal and distal enhancers and 9 μg of either empty expression vector or plasmid containing canine PXR). Transfected HepG2 cells were incubated at 37°C, 5% CO_2_ for 19–24 h. The cells were then trypsinized with TrypLE Express solution (Life Technologies) and either frozen in the presence of 10% DMSO for future use or seeded into clear bottom 96-well assay plate at a density of 5x10^4^ cells/well and incubated at 37°C, 5% CO_2_ for 24 h.

### Transactivation assays

For luciferase reporter assays involving transient transfectants, either freshly transfected cells or cells that have been stored in liquid N_2_ were seeded in 96-well plates as described above. For the rPXR stable cell line, cells were seeded into a 96-well plate at a density of 2x10^4^ cells/well. Following a 24 h recovery, transiently transfected cells or stably integrated rat PXR cells were treated with various chemicals dissolved in DMSO in triplicate wells. Phenobarbital was the only compound that was dissolved in sterile water. All compounds were diluted 1:1000 in culturing medium yielding 0.1% (v/v) final concentration of DMSO or water, therefore negative controls consisted of 0.1% DMSO or water. All treatments were performed for 48 h.

Assessment of cell viability and transcriptional activation was performed in the same wells using the CellTiter-Fluor cell viability assay and ONE-Glo Luciferase assay. Fluorescence was determined on a BioTek Synergy 2 Luminometer (BioTek Instruments, Winooski, VT) with excitation at 400 nm and emission at 510 nm. Following viability determination luminescence was monitored.

### Statistical analyses

Fluorescent light units were utilized to normalize for viable cell number in the luciferase (transcriptional activation) assay and expressed as the average of three determinations/chemical agent and concentration. The average luminescent units were divided by the average for solvent control to determine the fold activation. Data is expressed as mean ± standard error (SE) of three separate experiments performed in triplicate. The data was further normalized by dividing the values for each chemical by values obtained from assays with empty expression vector control. One-way multiple comparison ANOVA test in concert with Uncorrected Fisher’s LSD posttest was used to compare treatment groups with the negative controls. The significance threshold was set at p < 0.01. All statistical analyses were performed using Prism V6.0c (GraphPad, San Diego, CA). To determine receptor activation kinetics for positive controls, EC_50_ and E_MAX_ values were calculated using nonlinear regression analysis of typical log dose-response curves using Prism V6.0c (GraphPad, San Diego, CA).

## Results

### Comparison of canine PXR and CAR activation by various compounds

**Fig 1:**
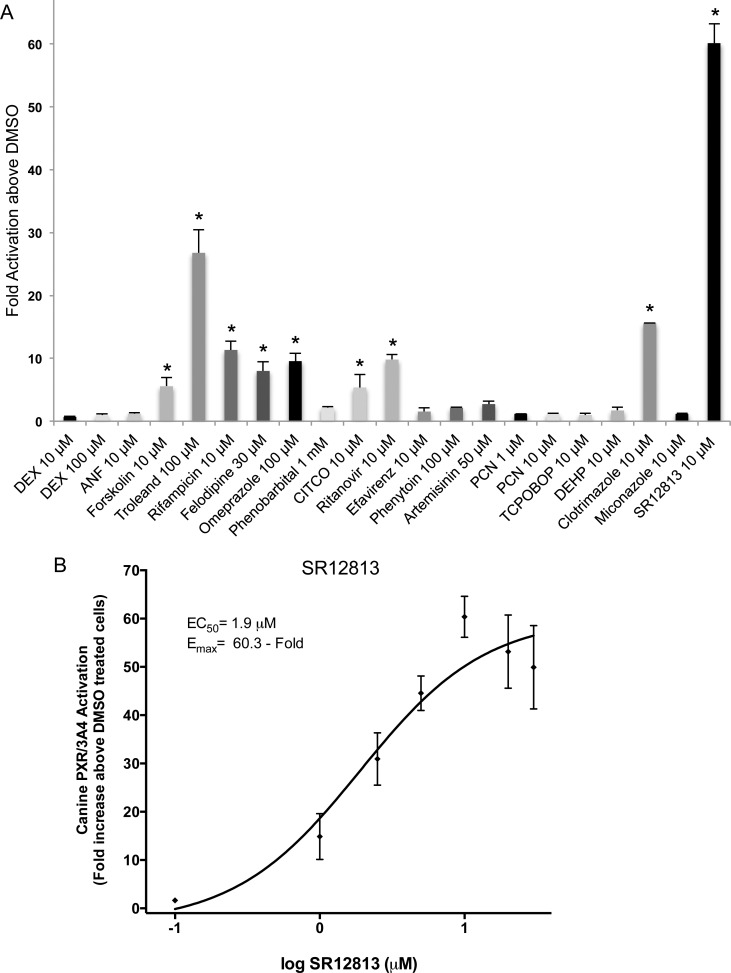
Effects of various compounds on transactivation of canine PXR. HepG2 cells were transfected with canine PXR or empty expression vector and luciferase reporter construct harboring the CYP3A4 proximal and distal promoter regions and seeded in 96-well assay plates as described in Materials and Methods. (A) Cells were treated with panel of 19 compounds for 48 h before detection of fluorescence for cell viability and luminescence for transcriptional activation. All luminescence values were normalized for cell viability. Data for each compound and concentration represents the mean ± SE from three independent experiments in triplicate expressed as fold activation above vehicle control treated cells. All data is normalized against fold activation values from transactivation assays with an empty expression vector. An asterisk denotes compounds exhibiting significant difference from their respective vehicle control at a level of p < 0.01. (B) The graph represents a SR12812 dose-response curve for canine PXR. Cells in triplicate wells were treated with 0.1, 1, 2.5, 5, 10, 20 and 30 μM SR12813 for 48h before luminescence was detected and normalized against cell viability (fluorescence values). Results are expressed as fold activation above 0.1% DMSO treated cells. Data represents the mean ± SE from three independent experiments in triplicate. EC_50_ and E_MAX_ values were calculated using nonlinear regression of a typical log dose-response curve.

**Fig 2:**
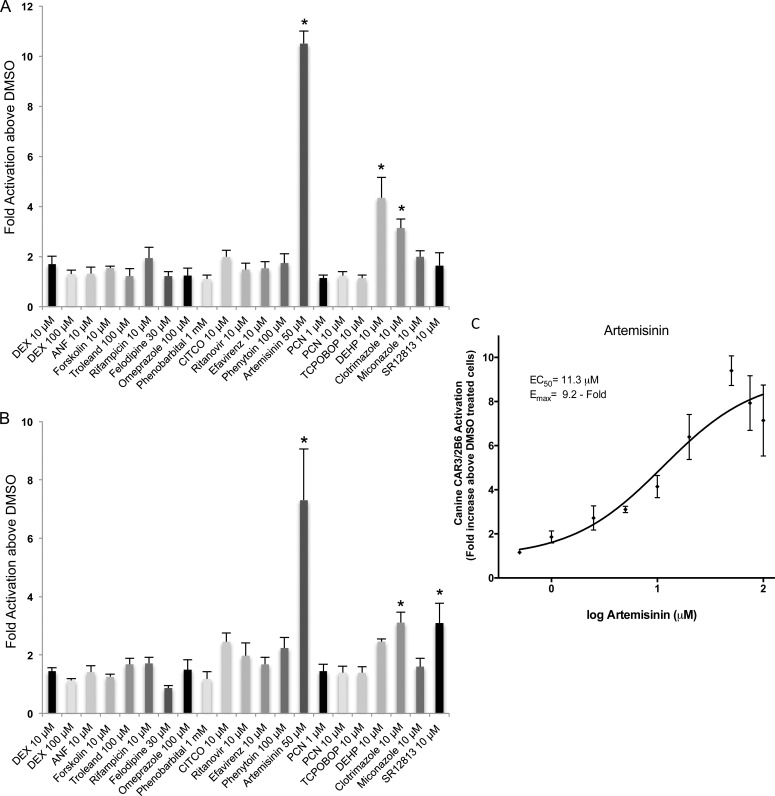
Transactivation of canine CAR3 by 19 different compounds. HepG2 cells were transfected with the canine CAR3 or empty expression vector and the CYP2B6 promoter and enhancer linked to a luciferase reporter vector (A) or luciferase reporter vector with CYP3A4 proximal and distal promoter regions (B) before seeding in 96-well plates as described in Materials and Methods. Cells were treated with the panel of 19 chemicals for 48 h before detection of fluorescence for cell viability and luminescence for transcriptional activation. All luminescence values were normalized for cell viability. Data for each test compound and concentration represents the mean ± SE from three independent experiments in triplicate expressed as fold activation above vehicle control treated cells. All data is normalized against fold activation values from transactivation assays with an empty expression vector. An asterisk denotes compounds exhibiting significant difference from their respective vehicle control at a level of p < 0.01. (C) A representative artemisinin dose-response curve for the cCAR3/2B6. Cells in triplicate wells were treated with 0.5, 1, 2.5, 5, 10, 20, 50, 75 and 100 μM artemisinin for 48 h before luminescence was detected and normalized against cell viability (fluorescence values). Results are expressed as fold activation above 0.1% DMSO treated cells. Data represents the mean ± SE from three independent experiments in triplicate. EC_50_ and E_MAX_ values were calculated using nonlinear regression of a typical log dose-response curve.

To identify receptor specificity, various compounds were tested for their ability to activate canine nuclear receptors, PXR (Fig 1A) and CAR3 (Fig 2A and 2B). Several compounds in this panel are known activators of human PXR, including rifampicin, omeprazole (OMP), tetraethyl 2-(3,5-di-*tert*-butyl-4-hydroxyphenyl)ethenyl-1,1-bisphosphonate (SR12813), phenobarbital, α -naphthoflavone (ANF), phenytoin, forskolin, ritanovir, and troleandomycin [12, 18, 20–22]. Of the 19 compounds examined, SR12813 (10 μM) exhibited the greatest canine PXR activation with a 60.1 ± 3.1 fold increase, followed by troleandomycin (100 μM), exhibiting a 26.8 ± 3.7 fold activation. Clotrimazole (10 μM), OMP (100 μM), and 10 μM rifampicin each produced 15.6 ± 0.07, 9.6 ± 1.3, and 11.3 ± 1.4 fold increases above DMSO treated cells, respectively (Fig 1A). Other canine PXR activators tested here that produced between 5 and 10- fold activation included ritanovir (10 μM), forskolin (10 μM), felodipine (30 μM), and CITCO (10 μM). The remaining compounds produced less than 5-fold activation, values not statistically different from DMSO. A dose-response curve with concentrations ranging from 0.1 to 30 μM was constructed for the most efficient canine PXR activator, SR12813 (Fig 1B). Because the activator produced a typical dose-response curve, an EC_50_ of 1.9 ± 0.2 μM and E_max_ of 60.3 ± 5.5 fold were obtained.

To determine whether canine PXR and CAR exhibited over-lapping ligand specificity, we examined the ability for the same panel of compounds to activate canine CAR3. In the transactivation assays we tested both the CYP2B6 (XREM-PBREM) response elements and the proximal and distal response elements from the CYP3A4 gene (Fig 2A and 2B). Given the highly conserved DNA binding domain of PXR and CAR across species, the response elements identified in the human genes were employed. When comparing the two different response elements we found that 50 μM artemisinin produced the greatest activation of the CAR3 receptor regardless of the promoter (Fig 2A and 2B). This agent produced slightly higher CAR3 activation with the CYP2B6 promoter (10.5 ± 0.5 fold) than with the CYP3A4 promoter (7.3 ± 1.8 fold). From remaining compounds examined, 10 μM clotrimazole and 10 μM SR12813 significantly activated canine CAR3 in the presence of the CYP3A4 promoter, 3.1 ± 0.4 and 3.1 ± 0.7 fold, respectively (Fig 2B). When the CYP2B6 promoter was employed, CAR3 activation was significantly activated by DEHP and clotrimazole at 10 μM, 4.3 ± 0.8 and 3.1 ± 0.4 fold, respectively (Fig 2A). The remaining compounds produced negligible CAR3 activation when either the CYP2B6 and CYP3A4 promoters were employed. A dose-response curve yielded EC_50_ and E_max_ values for artemisinin of 11.3 μM ± 1.4 and 9.2-fold ± 1.4 above DMSO, respectively, for cCAR3/2B6 (Fig 2C). When the CYP3A4 promoters (cCAR3/3A4), were employed artemisinin produced an atypical dose-response curve (data not shown). The primary difference between the two enhancers was that DEHP produced significant activation only when the CYP2B6 promoter was used and SR12813 exhibited activation only in the presence of the CYP3A4 promoter (Table 1).

**Table 1:** Compounds Activating One or More Nuclear Receptors.

Agent	Concentration	Nuclear Receptor (Fold Activation^a^)			
	μM	Canine PXR	Canine CAR3	Rat PXR	Rat CAR3
Dexamethasone	10	-^b^	-	60.9 ± 8.4	-
	100	-	-	59.9 ± 12.7	-
ANF	10	-	-	-	3.4 ± 0.8^c^
Forskolin	10	5.6 ± 1.4	-	5.8 ± 0.5	-
Troleandomycin	100	26.8± 3.7	-	-	-
Rifampicin	10	11.3 ± 1.4	-	-	-
Felodipine	30	8.0 ± 1.5	-	11.5 ± 1.3	-
Omeprazole	100	9.6 ± 1.3	-	9.8 ± 0.9	-
CITCO	10	5.4 ± 2.1	-	-	-
Ritanovir	10	9.8 ± 0.8	-	5.1 ± 1.1	-
Efavirenz	10	-	-	7.5 ± 2.3	3.9 ± 0.6^c^ 7.4 ± 3.3^d^
Artemisinin	50	-	10.5 ± 0.5^c^ 7.3 ± 1.8^d^	-	7.1 ± 0.4^c^ 10.5 ± 2.2^d^
Pregnenolone 16α carbonitrile	1	-	-	11.7 ± 0.6	-
	10	-	-	10.4 ± 0.7	-
DEHP	10	-	4.3 ± 0.8^c^	-	-
Clotrimazole	10	15.6 ± 0.07	3.1 ± 0.4^c^ 3.1 ± 0.4^d^	12.5 ± 0.9	13.7 ± 0.8^c^ 26.9 ± 1.3^d^
Miconazole	10	-	-	9.1 ± 1.2	5.1 ± 1.0^c^ 5.9 ± 1.9^d^
SR12813	10	60.1 ± 3.1	3.1 ± 0.7^d^	6.1 ± 0.6	-

^a^ Values represent the fold activation above DMSO treated cells normalized to the cell number in each well. Each value is the mean of 3 or more determinations and is expressed as fold increase ± SE at p < 0.01.

^b^ Indicates that there was no statistically significant difference from control (DMSO treated cells) at p < 0.01.

^c^ Fold activation of CAR3 in the presence of the CYP2B6 promoter and enhancer

^d^ Fold activation of CAR3 in the presence of the CYP3A4 promoter and enhancer

### Comparison of rat PXR and CAR activation by 19 compounds

**Fig 3:**
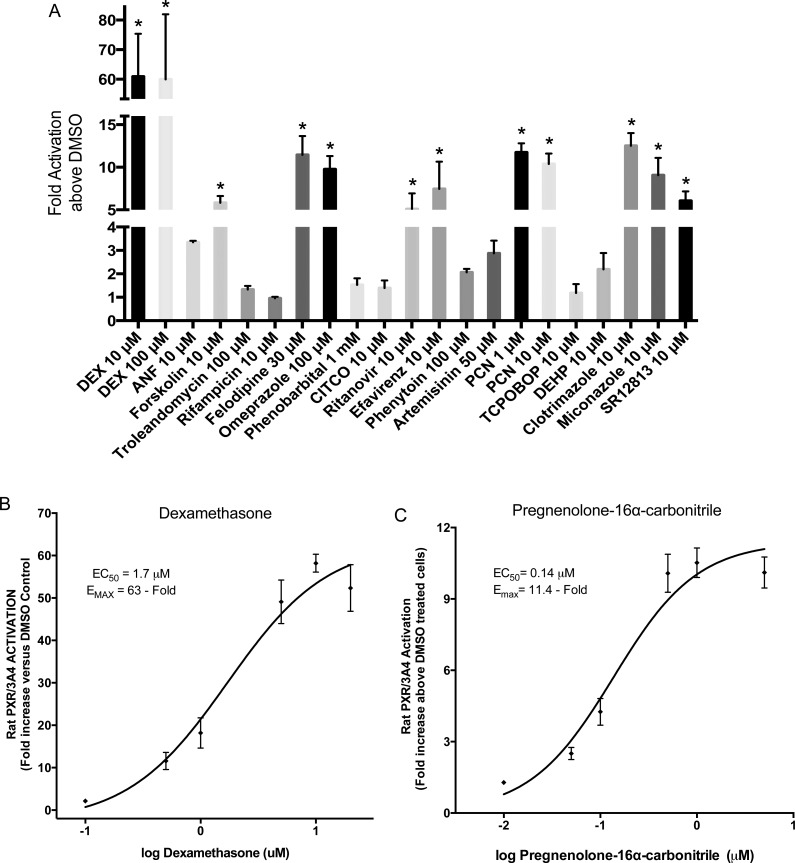
Rat PXR transactivation by 19 compounds in a stable cell line. A rat PXR stable cell line over-expressing rat PXR and a luciferase reporter vector containing the CYP3A4 proximal and distal enhancer regions [14] was used to evaluate activation of rat PXR by various compounds (A). rPXR cells were seeded in 96-well plates and treated with the 19 compounds for 48 h before fluorescence (cell viability) and luminescence (transcriptional activation) detection. All luminescence values were normalized for cell viability. The data represent the mean ± SE from three independent experiments in triplicate expressed as fold activation above vehicle control treated cells. An asterisk denotes compounds exhibiting significant difference from their respective vehicle control at a level of p < 0.01. (C-D) Dose-response curves for two rat PXR positive controls, Dexamethasone (B) and Pregnenolone 16α- carbonitrile (C). Concentrations were 0.1, 0.5, 1, 5, 10 and 20 μM for Dexamethasone (B) and 0.01, 0.05, 0.1, 0.5, 1 and 5 μM for Pregnenolone 16α- carbonitrile (C). Results are expressed as fold activation above 0.1% DMSO treated cells. Data represents the mean ± SE from three independent experiments in triplicate. EC_50_ and E_MAX_ values were calculated using nonlinear regression of typical log dose-response curves.

To determine if PXR is the primary receptor activated by more agents and to a greater extent in the rat, we examined the same panel of compounds employing cell lines containing rat PXR or CAR3. Dexamethasone (10 μM) was the most significant activator of rat PXR and produced a fold increase over DMSO of 60.9 ± 8.4 (Fig 3A). Increasing the concentration to 100 μM did not increase the fold activation (59.9 ± 12.7). A dose-response curve with concentrations ranging between 0.1 and 20 μM, was constructed and provided an EC_50_ of 1.7 ± 0.14 μM with an E_max_ of 63-fold ± 4.3 (Fig 3B). Of the compounds tested in the panel of 19, 10 exhibited a significantly higher luciferase activity when compared to DMSO treated cells (Table 1). Apart from dexamethasone, 1 μM pregnenolone 16 α-carbonitrile (PCN) exhibited the greatest fold-increase over DMSO (11.7 ± 0.6 fold) with 10 μM PCN producing a 10.4 ± 0.7 fold increase (Fig 3A, Table 1). Despite the higher E_max_ value produced by dexamethasone (63 ± 4.3 fold), a dose-response curve revealed that PCN was a more potent activator of rat PXR exhibiting an EC_50_ value of 0.14 μM ± 0.02 vs 1.7 μM ± 0.14 produced by dexamethasone (Fig 3B and 3C). Other agents that significantly activated rat PXR at the 10 μM concentration included, clotrimazole, miconazole, SR12813, forskolin, efavirenz, and ritanovir with values of 12.5 ± 0.9, 9.1 ± 1.2, 6.1 ± 0.6, 5.8 ± 0.5, 7.5 ± 2.3, and 5.1 ± 1.1 fold increase, respectively (Fig 3A). At higher concentrations of certain compounds, OMP (100 μM) and felodipine (30 μM) significantly activated rat PXR to 9.8 ± 0.9 and 11.5 ± 1.3 fold above DMSO, respectively (Fig 3A). The remaining compounds, ANF (10 μM), troleandomycin (100 μM), rifampicin (10 μM), phenobarbital (1 mM), CITCO (10 μM), phenytoin (100 μM), artemisinin (50 μM), TCPOBOP (10 μM), and DEHP (10 μM) failed to elicit significant activation of the rat PXR (Fig 3A).

**Fig 4:**
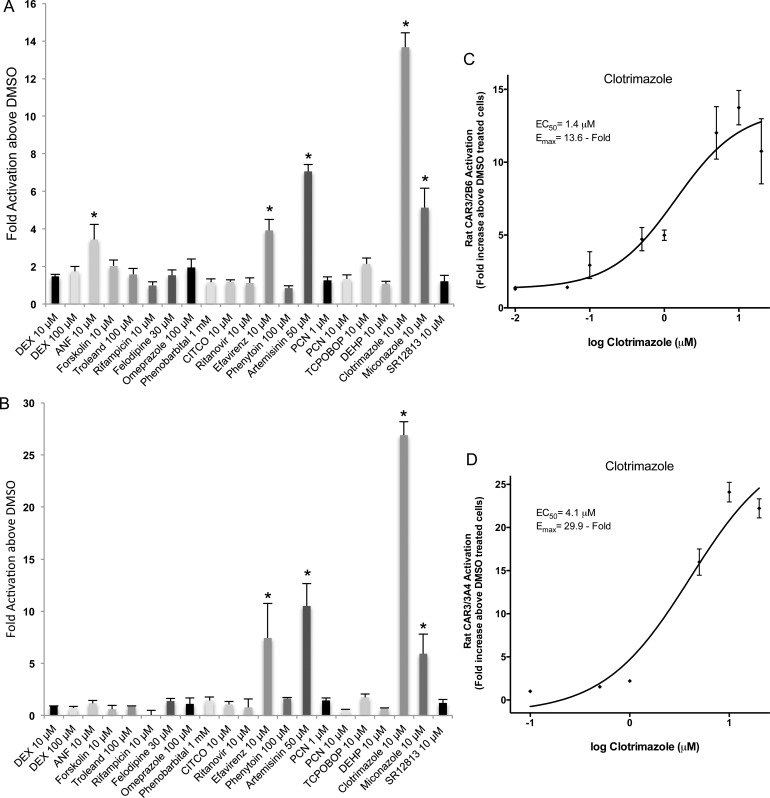
Effects of various compounds on transactivation of rat CAR3. HepG2 cells were transfected with rat CAR3 or empty expression vector and the CYP2B6 promoter and enhancer linked to a luciferase reporter vector (A) or luciferase reporter vector with CYP3A4 proximal and distal promoter regions (B) before seeding in 96-well plates as described in Materials and Methods. Cells were treated with the same panel of chemicals for 48 h before detection of fluorescence and luminescence. All luminescence values were normalized for cell viability. Data represents the mean ± SE from three independent experiments in triplicate expressed as fold activation above vehicle control treated cells. All data is normalized against fold activation values from transactivation assays with an empty expression vector. An asterisk denotes significant difference from vehicle controls at a level of p < 0.01. (C-D), Representative clotrimazole dose-response curves for rCAR3/2B6 (C) and rCAR3/3A4 (D). Cells in triplicate wells were treated with 0.01, 0.05, 0.1, 0.5, 1, 5, 10 and 20 μM clotrimazole (C) or 0.1, 0.5, 1, 5, 10 and 20 μM clotrimazole (D) for 48 h before luminescence was detected and normalized against cell viability. Results are expressed as fold activation above 0.1% DMSO treated cells. Data represent the mean ± SE from three independent experiments triplicate. EC_50_ and E_MAX_ values were calculated using nonlinear regression of a typical log dose-response curve.

Similar to the canine CAR3 studies, two different CYP promoter regions, XREM and PBREM of the CYP2B6 gene (rCAR3/2B6) and the proximal and distal enhancers of CYP3A4 (rCAR3/3A4) were transfected with rat CAR3 and exposed to the panel of 19 compounds. Several known human CAR direct activators, including CITCO and artemisinin, and indirect activators, such as phenobarbital, efavirenz, and phenytoin were tested to determine if similarities to the human or dog receptors existed. Moreover, compounds were compared to rat PXR activators. From the set of compounds tested using rCAR3/2B6, only 5 of the 19 compounds produced activation (Fig 4A). At 10 μM ANF, efavirenz, clotrimazole and miconazole exhibited significant transactivation of rat CAR3 to 3.4 ± 0.8, 3.9 ± 0.6, 13.7 ± 0.8, and 5.1 ± 1.0 fold above DMSO, respectively (Fig 4A and Table 1). At 50 μM, artemisinin also significantly activated rCAR3/2B6 to 7.1 ± 0.4 above control (Fig 4A). None of the other compounds tested produced significant activation of rCAR3/2B6 (Fig 4A). When the CYP3A4 promoter and enhancer were utilized, only 4 agents significantly activated rat CAR3, clotrimazole (10 μM) exhibiting the most significant fold activation reaching 26.9 ± 1.3 fold above DMSO treated cells (Fig 4B). Artemisinin (50 μM) significantly activated rCAR3/3A4 producing a 10.5 ± 2.2 fold increase (Fig 4B) which was slightly higher than that produced by rCAR3/2B6 (Table 1). Efavirenz (10 μM) and miconazole (10 μM) also exhibited rCAR3/3A4 activation with 7.4 ± 3.3 fold and 5.9 ± 1.9 increases above DMSO treated cells, respectively (Fig 4B, Table 1). Interestingly, all test agents producing a significant activation, exhibited higher activation when the CYP3A4 promoter and enhancer was employed (Table 1). Because clotrimazole produced the greatest fold activation of rCAR3/2B6 and rCAR3/3A4, dose-response curves were constructed to assess potency (Fig 4C and 4D). Typical dose-response curves with concentrations ranging from 0.01 μM to 20 μM (rCAR3/2B6) or 0.1 μM to 20 μM (rCAR3/3A4) were generated and from the curves we obtained an EC_50_ of 1.4 μM ± 0.3 and an E_max_ of 13.6 ± 2 fold for rCAR3 activation of the CYP2B6 promoter/enhancer (Fig 4C). When the CYP3A4 promoter was employed the EC_50_ value was only slightly higher, 4.1 μM ± 0.6, but the E_max_ was more than twice as high, 29.9 fold ± 0.9 above DMSO treated cells (Fig 4D).

## Discussion

Cell-based reporter assays facilitate the identification of chemicals activating NRs, including those from human, rabbit, rat, mouse, monkey, pig, zebrafish, and other species [23]. Results from such assays suggest that PXR and CAR from various species may have evolved to have different functions, such as the promiscuousness of PXR and high basal activity of CAR, while sharing some similarities [23]. With regards to functionality, differences that exist in ligand-specificity between species have been well documented. However, comparisons between CAR activators and those of PXR have largely been ignored primarily due to a lack of availability of adequate CAR test systems. One reason for the small number of assays available to assess CAR activation may be that several alternative splice variants of CAR are concurrently expressed in liver that exhibit different ligand specificities [24–26]. Another reason is that transient transfection of CAR in tumor cells normally results in spontaneous nuclear translocation with subsequent high constitutive activity [27–30]. This makes identification of CAR activators challenging and to date, a relatively limited number of CAR modulators have been reported. In an attempt to overcome this challenge, one splice variant, hCAR3, that contains an in-frame insertion of five amino acids (APYLT) in the LBD of wild-type (wt) CAR (hCAR1), was found to produce lower constitutive activity allowing xenobiotic activation to be assessed [31, 32]. This has greatly facilitated the development of cell-based reporter gene assays, including assays to assess CAR activation in various species [17]. Full-length CAR3 variants from mouse, rat and dog maintained species specificity in ligand binding and molecular modeling verifying that the 5 amino acid insertion present in CAR3 results in a receptor with virtually an identical LBD to that of wt CAR [17]. This indicates that “humanized CAR3”, i.e. species-specific wt CAR with the human CAR3 signature may serve as a unique surrogate for wt CAR where ligand specificity is to be determined. We utilized this approach here to compare the ligand-specificity for rat and canine CAR receptors employing compounds implicated in the activation of human, rat and mouse CAR.

The compounds selected for these studies reflect species-specific CAR activation stemming from variations in the LBD of the NR or because a single compound may have the opposite species-specific effect on the receptor. For example, CITCO is a prototypical human CAR activator [33] while 1,4-bis[2-(3,5-dichloropyridyloxy)]benzene (TCPOBOP) is selective for mouse CAR [34]. Clotrimazole binds to CAR from most species, but functions as a human CAR inverse agonist and an agonist for mouse and rat CAR [17, 35–37]. In the studies presented here, several known human CAR direct activators, including CITCO and artemisinin, along with indirect activators, phenobarbital, efavirenz, and phenytoin [32, 33, 38–40] were tested to demonstrate species-specificity of canine and rat CAR3. DEHP, a plasticizer that activates PPARα, was also selected because it may activate CAR in human hepatocytes and mouse livers [41, 42]. However, identifying which NR, PXR or CAR, is activated in primary hepatocytes by DEHP is complicated because of cross-talk between the receptors for the DNA response elements.

Cross-talk between hCAR and hPXR reflects an efficient and coordinated mechanism in defending the human body from xenobiotic challenges [1, 43]. However, this feature also generates difficulties in defining the exact biological function of these receptors. CAR binds to both proximal and distal (XREM) CYP3A response elements [30, 34, 44–47] and PXR binds to DR-4 type NR1 and NR2 sequences present in the CYP2B PBREM region [48–50]. This cross-talk feature was exhibited in the present investigation by use of human DNA binding element. We employed both the XREM-PBREM response elements from the prototypical CAR promoter, CYP2B6 [16] as well as proximal (PXRE) and distal (XREM) response elements from CYP3A4 promoter [18] (Figs 2A, 2B, 4A and 4B). Human response elements, CYP3A4 and CYP2B6 and animal homologs of these elements exhibit a high degree of conservation. This conservation is also present in the DNA binding domain of the receptor which is typically greater than 95% homologous [8]. The ability to utilize different promoters/enhancers in the assays described here is an advantage over other systems because the best pair of a NR and a response element can be selected for optimal results. Furthermore, employing response elements from human genes for assessing activation of NRs from other species indicates that this system is highly efficient based on the high levels of transactivation by compounds witnessed here. Indeed, ligand-binding assays, such as fluorescence polarization, time-resolved FRET and mammalian two hybrid systems employ only the LBD of the receptor and in the case of the two hybrid system a GAL4 promoter from yeast and a DNA region that binds to an upstream activating sequence (UAS) are utilized for assessing species specificity. These assays are very useful tools for identifying species specific ligands particularly during early drug discovery [21], but it can be argued that utilizing human response elements and a full-length receptor in our species-specific transactivation assays provides a distinct advantage over these systems in data extrapolation to *in vivo* [14, 20, 21].

When comparing the two separate promoter and enhancer regions, we found that for all four assays systems, 50 μM artemisinin and 10 μM clotrimazole activated CAR3 (Table 1). These results are similar to those previously described [17]. For canine CAR3, artemisinin proved to be the better activator while clotrimazole was the best activator of rat CAR3 regardless of the promoter and enhancer (Figs 2A, 2B, 4A and 4B and Table 1). In contrast, canine CAR3 was activated by 10 μM SR12813 only in the presence of the CYP3A4 promoter (Fig 2B) but 10 μM DEHP significantly activated only canine CAR3/2B6 (Fig 2A), a finding that has not been described previously. A similar trend was observed for the rat CAR3 where ANF activated this receptor only in the presence of the CYP2B6 promoter and produced a negligible effect when the CYP3A4 promoter and enhancer were employed (Table 1). Differences were also observed in the extent of activation among the two promoters and enhancers; greater activation by clotrimazole was observed for rCAR3/3A4 than rCAR3/2B6 (Table 1). Indeed, all compounds activating rat CAR3 produced greater activation when the CYP3A4 promoter and enhancer were employed with the exception of ANF, suggesting that although somewhat lower activation was observed for CYP2B6 and CAR3, more compounds were activated in the presence of this pair. In contrast, artemisinin and clotrimazole produced similar activation of canine CAR3 regardless of the promoter and enhancer. The fewer compounds activating rat CAR3 when the CYP3A4 promoter was employed may be due to the ER6 motif of PXRE which does not contain DR4 type NR1 and NR2 sequences as does the PBREM region of CYP2B6 promoter and enhancer [48–50]. This suggests that CAR3 might bind more efficiently to the CYP2B6 promoter than the CYP3A4 promoter. However, this does not explain the results obtained with the canine CAR3 and the CYP3A4 and CYP2B6 promoters where an equal number of compounds activated the receptor. Overall, the exact reason for different responses between the two P450 promoters is unclear but illustrates the inherent difficulty of using test systems such as primary cultures to elucidate the mechanisms involved in induction of P450 enzymes. Of the 19 agents tested here for activation of rat or dog PXR and CAR3, 9 compounds activated dog PXR and 10 were capable of activating rat PXR (Figs 1A and 3A and Table 1). In contrast, only 4 of the agents tested activated dog CAR3 and 5 activated rat CAR3 (Figs 2A and 4A, Table 1). The three agents that activated rat CAR3/2B6 but not dog CAR3/2B6 were efavirenz, ANF, and miconazole, which constitutes a novel finding (Table 1). When comparing PXR and CAR3 from both species, the only agent that activated both receptors, albeit to different extents, was clotrimazole (Table 1). This drug exhibits broad range of specificity as it has previously been shown to activate human, rat, mouse, and canine PXR [14, 18, 23], confirming our results. Comparison of rat PXR and CAR revealed that efavirenz, clotrimazole and miconazole produced activation of both receptors (Table 1) whereas, dexamethasone only activated rat PXR but had no effect on rat CAR3 (Figs 3A and 4A and 4B). Activation of rat PXR by clotrimazole and miconazole has been described previously [14] and results were similar to those shown here.

Other differences between CAR3 and PXR activation were noted. First, the fold activation for compounds other than clotrimazole, exhibiting significant differences from control (DMSO-treated) cells is much greater for PXR than for CAR3 (Table 1). The most potent activators of CAR3 produced only 3 to 27—fold activation above DMSO treated cells while those of PXR produced between 5–60 fold. Second, there are more compounds exhibiting statistically significant differences when compared to control when PXR is the nuclear receptor (Table 1). This is not surprising given that a recent report [51], using cell-based HTS assays to identify novel human CAR agonists and antagonists showed that of 2816 compounds in the NIH Chemical Genomics Center Pharmaceutical Collection library, 115 were agonists and 152 were antagonists, demonstrating the low number of compounds that ligand to human CAR. We would anticipate similar findings for rat and canine CAR. In contrast, screening for PXR agonists from the same library identified 750 compounds that displayed activation of human PXR and 512 that activated both rat PXR and human PXR [12]. In the present study of compounds that significantly activated canine PXR (Fig 1A), only SR12813, rifampicin and clotrimazole have been reported previously as canine PXR activators with SR12813 exhibiting the most significant activation [23], consistent with our results (Table 1). Importantly, activation of canine PXR by forskolin, troleandomycin, ritanovir, omeprazole, CITCO and felodipine is a novel finding. Compounds activating rat PXR that have not previously been reported include felodipine, omeprazole, ritonavir and efavirenz (Table 1). Our results demonstrate the ability of PXR to accommodate a much more diverse population of xenobiotics in its ligand binding region whereas CAR3 has a much more limited ability to bind xenobiotics. These results demonstrate further the importance of employing systems that only contain a single nuclear receptor to identify the receptor responsible for any potential DDIs.

When comparing results from canine and rat PXR transactivation assays, considerable differences were observed. For example, troleandomycin, rifampicin, and CITCO activate canine PXR, but not rat PXR. Dexamethasone, miconazole, and PCN activate rat PXR, but not canine PXR (Figs 1A and 3A). Overlapping function was also observed. Forskolin, felodipine, OMP, ritonavir, SR12813 and clotrimazole activated both, canine and rat PXR (Figs 1A and 3A). We also identified compounds that overlap in activation capabilities between canine and human PXR including rifampicin, felodipine, troleandomycin, omeprazole, CITCO, ritonavir and SR12813 [12, 18, 20–22]. However, differences were also observed. Phenobarbital and phenytoin have been shown to activate human PXR [11, 18, 37] but failed to elicit a significant activation of canine or rat PXR (Figs 1A and 3A). It is well known that phenobarbital increases the expression of rat and dog cyp3as and cyp2bs [52–54], but our results were unable to delineate which NR was involved in this induction. In a similar system, phenobarbital produced substantial activation of hPXR [18] but not rat PXR [14], so it is not surprising that this agent had a negligible effect on rat PXR. It is possible that phenobarbital activates NRs by ligand independent pathways that may be absent in these cell systems.

Recently, it was determined that human and mouse CAR are regulated by phosphorylation. When the receptor is phosphorylated at Thr^38^ [55–59] via epidermal growth factor receptor (EGFR) signaling, CAR is sequestered in the cytoplasm [60]. Phenobarbital and possibly other indirect activators bind to EGFR preventing its interaction with epidermal growth factor, thereby repressing its function and decreasing phosphorylation, allowing CAR to translocate to the nucleus and producing activation of target genes. Results presented here suggest that some indirect activators, such as phenobarbital may not activate canine and rat CAR3, consistent with previously published findings [17]. One explanation may be that phenobarbital may activate a different variant of CAR other than the CAR3 employed here or different/multiple NRs in these species. With regards to the human CAR variants, it has been shown that indirect activators, such as phenobarbital, efavirenz and phenytoin activate CAR1 [39], suggesting that a different CAR variant may lead to activation of the canine or rat receptor by phenobarbital or phenytoin.

The goal of this investigation was to develop species-specific cell-based transactivation assays to identify NME’s with the ability to activate canine and rat PXR and CAR. We demonstrated the species-specificity, shown that PXR and CAR ligands differ, and have established potent and suitable positive controls for each assay; SR12813 for canine PXR (Fig 1B), artemisinin for canine CAR3 (Fig 2C), PCN for rat PXR (Fig 3C) and clotrimazole for rat CAR3 (Fig 4C and 4D). To the best of our knowledge, there have been only one other study that examined rat and dog CAR [17] and two other studies that examined rat PXR [11, 14]. Results from the investigations described here were similar to those in the three studies. However, different from those previously published, many more compounds were employed in the studies described here and several unique activators were identified. One additional study examined dog PXR [23]. However, the latter study utilized only the ligand-binding domain of the receptor and examined very few compounds, making direct comparisons to our studies difficult, given that we utilized the full-length receptor. Few advances have been made in establishing *in vitro* assays that can determine canine PXR activators. Importantly, this is the first investigation to compare PXR and CAR activators in a single study for these two species. [14, 17]

The significance of employing assays such as those described here for assessing canine and rat PXR and CAR activation is several fold. First, the number of animals utilized for toxicology studies can be decreased. Second, selecting an appropriate animal species for toxicology testing in preclinical drug safety trials which has demonstrated to be challenging, [61] may be facilitated by *in vitro* studies such as those described here. Furthermore, ligand specificity of NRs from different species can lead to alterations in biotransformation causing inaccurate predictions due to auto-induction. Employing the NR assays prior to animal treatment would allow identification of auto-induction that could potentially alter the outcome of pharmacokinetics *in vivo*. Finally, these assays would be valuable for the drug discovery process in selecting nonhuman pharmacological model systems [21]. In summary, we demonstrate that canine and rat PXR and CAR3 transactivation assays are useful for medium and high throughput screening of species-specific PXR and CAR activators. Furthermore, these assays allow for a more definitive identification of the NR involved in the induction of a particular P450 enzyme and in predicting potential adverse drug effects.

## Abbreviations

ANF: α-naphthoflavone
CAR: constitutive androstane receptor
cCAR3/2B6: combination of canine CAR3 and the human CYP2B6 promoter and enhancer
cCAR3/3A4: combination of canine CAR3 and human CYP3A4 promoter and enhancer
CITCO: 6-(4-Chlorophenyl)imidazo[2,1-b][1,3]thiazole-5-carbaldehyde O-(3,4-dichlorobenzyl)oxime
DDIs: drug-drug interactions
DEHP: bis (2-ethylhexyl) phthalate
DEX: dexamethasone
DMSO: dimethyl sulfoxide
EGFR: epidermal growth factor receptor
hCAR: human CAR
hPXR: human PXR
NMEs: new molecular entities
NR: nuclear receptor
OMP: omeprazole
PBREM: phenobarbital-responsive enhancer module
PCN: pregnenolone-16 α –carbonitrile
PPAR: peroxisome proliferator activated receptor
PXR: pregnane X receptor
PXRE: pregnane X responsive element
rCAR3: rat CAR3
rCAR3/2B6: combination of rat CAR3 and the human CYP2B6 promoter and enhancer
rCAR3/3A4: combination of rat CAR3 and human CYP3A4 promoter and enhancer
rPXR: rat pregnane X receptor
SR12813: tetraethyl 2-(3,5-di-*tert*-butyl-4-hydroxyphenyl)ethenyl-1,1-bisphosphonate
TCPOBOP: 1,4-Bis-[2-(3,5-dichloropyridyloxy)]benzene, 3,3′,5,5′-Tetrachloro-1,4-bis(pyridyloxy)benzene
wt: wild-type
XREM: xenobiotic responsive enhancer module
